# Single domain based bispecific antibody, Muc1-Bi-1, and its humanized form, Muc1-Bi-2, induce potent cancer cell killing in muc1 positive tumor cells

**DOI:** 10.1371/journal.pone.0191024

**Published:** 2018-01-22

**Authors:** Yumei Li, Changhua Zhou, Jing Li, Jiayu Liu, Limin Lin, Li Li, Donglin Cao, Qing Li, Zhong Wang

**Affiliations:** 1 School of Pharmaceutical Sciences, School of Pharmaceutical Sciences, Guangzhou, China; 2 Center for Cellular & Structural Biology, Sun Yat-sen University, Guangzhou, China; 3 Department of Laboratory Medicine, Guangdong Second Provincial General Hospital, Guangzhou, China; Northwestren University, UNITED STATES

## Abstract

Muc1 is one of the most studied tumor antigens. However, antibodies or antibody-toxin conjugates against Muc1 have not shown significant efficacy for tumors with Muc1 overexpression. In this study, we employed bispecific antibody approach to target Muc1 positive tumor cells. A novel bispecific antibody, Muc1-Bi-1, was constructed by linking single domain antibodies, anti-Muc1-VHH and anti-CD16-VHH. Muc1-Bi-2, the humanized form of Muc1-Bi-1, was also constructed by grafting. Both Muc1-Bi bispecific antibodies can be efficiently expressed and purified from bacteria. In vitro, the Muc1-Bi bispecific antibodies can recruit Natural Killer (NK) cells to drive potent and specific cell killing of Muc1-overexpressing tumor cells. In xenograft model, the Muc1-Bi bispecific antibodies can suppress tumor growth in the presence of human peripheral blood mononuclear cells (PBMC). These data suggested that the single domain based Muc1-Bi may provide a valid strategy for targeting tumors with Muc1 overexpression.

## Introduction

The advancement of protein engineering has opened opportunities to generate specific functions of proteins, including bispecific molecules [1, 2]. Bispecific antibodies combine specificities of two antibodies and simultaneously target two different antigens or epitopes. With the functions conferred by two different antibodies, bispecific antibodies can interfere with a variety of surface receptors or ligands, and are actively studied for many diseases, especially in cancer therapy by recruiting immune cells to directly target and kill tumor cells [3–6].

Muc1 is one of the most studied tumor antigens [7]. Muc1 belongs to the membrane-bound class of Mucins, which are type I membrane proteins with single transmembrane domains and different lengths of cytoplasmic tail at the C-terminus [8]. Muc1 is a highly glycosylated protein with O-linked carbohydrates to Serines and Threonines within the variable number of tandem repeats (VNTR) region [9, 10], which has anywhere between 20 to 120 or more repeats composed of 20 amino acids [11]. Muc1 is normally expressed at low levels on the apical surface of most glandular epithelial cells [12], which loses polarity and highly upregulated during tumorigenesis [13]. The aberrant Muc1 expression occurs in many types of human cancers including colon, lung, pancreas, breast, ovarian, prostate, kidney, stomach and head and neck cancers [14–16].

The role of Muc1 in tumorigenesis is still not well understood [17]. As a broadly expressed tumor antigen, Muc1 presents as an ideal target for tumor therapy. However, targeting Muc1 by antibodies is complicated by its long VNTR repeats and glycosylation. For example, a panel of monoclonal anti-Muc1 antibodies showed various binding properties against Muc1[18], likely due to the different levels of Muc1 expression, glycosylation, and VNTR repeats. Antibodies raised against Muc1 from normal tissues have failed in clinical development [19]. Recently, antibodies generated based on the glycosylation differences of normal and tumor Muc1 have been advanced into clinical with promising efficacy. For example, Pankomab-GEX, a humanized antibody targeting the tumor glycosylated Muc1, has showed good responses in patients by inducing antibody-dependent cell-mediated cytotoxicity (ADCC)[20]. Recently, chimeric antibody receptor T cell (CAR-T) immunotherapy also showed promises for Muc1 high expression tumors [21].

Natural killer (NK) cells are important innate immunity cells by recognizing infected cells or cells stressed by malignant transformation [22]. In antibody mediated targeted cancer therapy, such as Herceptin, or Rituximab, NK cells are the major players of the antibody-dependent cell-mediated cytotoxicity (ADCC). To mediate direct cytotoxicity of NK cells to tumor cells, bispecific antibodies engaging NK cells have also been investigated [23]. In this study, we constructed a novel bispecific antibody, Muc1-Bi, by linking single domain antibodies, anti-Muc1 and anti-CD16. The Muc1-Bi bispecific antibody can recruit NK cells to drive potent cancer cell killing in Muc1-overexpression cancer cells, providing a valid alternative for cancer therapy.

## Materials and methods

### Construction, expression, and purification of Muc1-Bi bispecific antibodies

**Fig 1:**
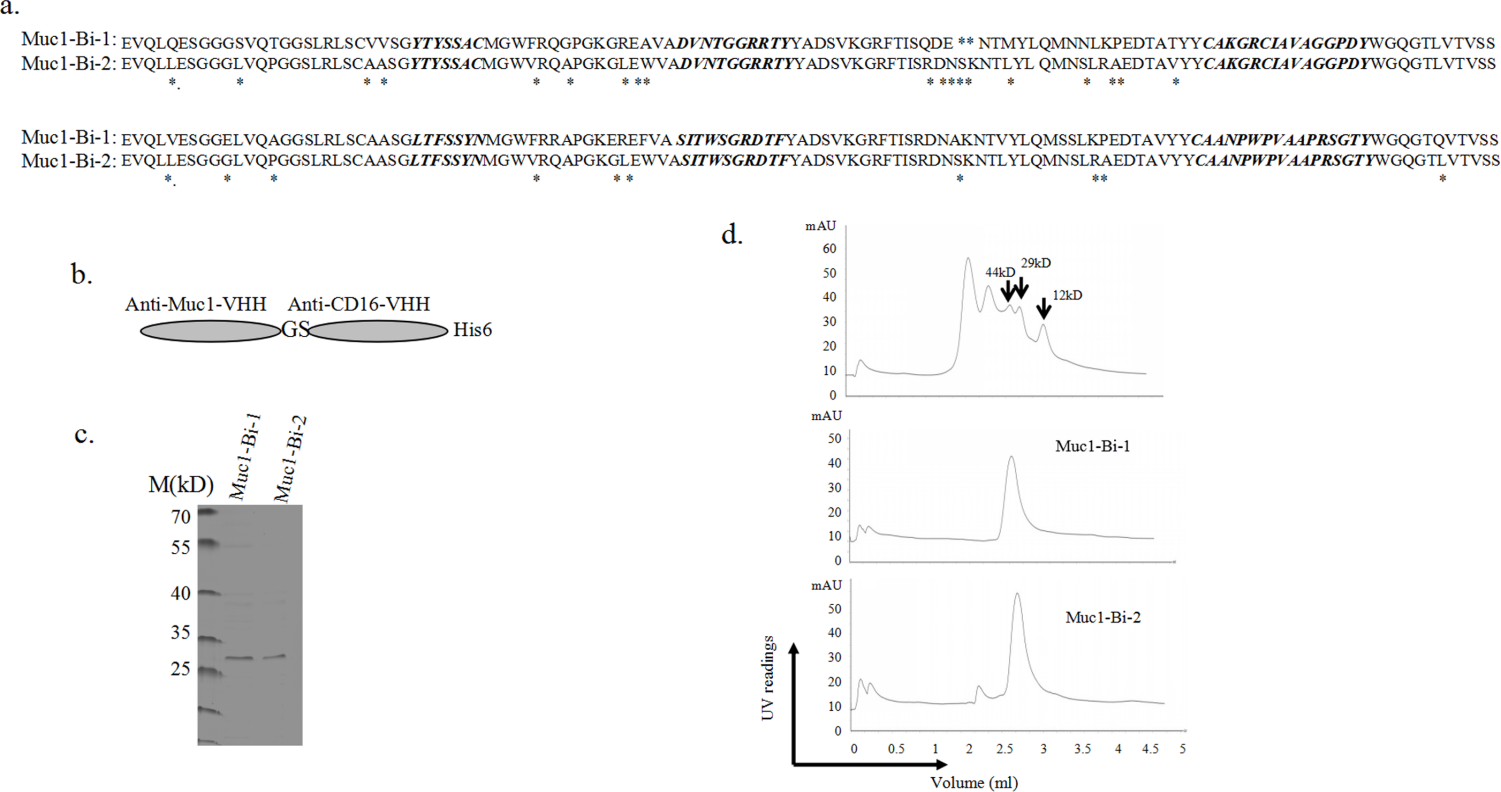
Expression and purification of the Muc1-Bi-1 and Muc1-Bi-2 from *E*. *coli*. a). The sequences of Muc1-Bi-1 and humanized Muc1-Bi-2. The CDRs of Muc1-VHH and CD16-VHH was grafted to DP-47 reference framework and only a few amino acids were changed (shown as *). b). The diagram of Muc1-Bi-1 and Muc1-Bi-2 constructs. To facilitate protein detection and purification, a His_6_-tag was added to the c-terminal end. Two amino acids GS were added between anti-Muc1 and anti-CD16 VHHs. c). Coomassie blue–staining of purified proteins (Muc1-Bi-1 or Muc1-Bi-2) after Ni-NTA affinity chromatography. d). Size exclusion chromatography of protein marker (top panel), Muc1-Bi-1 (middle panel), and Muc1-Bi-2 (bottom panel).

The Muc1-Bi-1 bispecific antibody was constructed by linking 2 single domain antibodies, anti-Muc1-VHH (GenBank: FJ799116.1) and anti-CD16-VHH [23] (Fig 1A) by gene synthesis (Genscript) and cloned into the pET21a or pET26b plasmid. A histidine tag was added to the carboxyl terminus for detection and purification.

Muc1-Bi-2 bispecific antibody was the humanized Muc1-Bi-1. Humanization was performed by grafting the CDRs of Muc1-VHH and CD16-VHH to DP-47 reference framework [24].

To purify Muc1-Bi bispecific antibodies, cytoplasmic protein purification was performed. Briefly, Muc1-Bi expression were transformed into BL21(DE3) competent cells containing 100 μg/mL Amp. When the cell culture reached an OD_600_ of 0.8–1.0, 0.1mM isopropyl β-1-ithiogalactopyranoside (IPTG) was added to induce protein expression. The Muc1-Bi bispecific antibodies were then purified by immobilized Ni-NTA affinity chromatography as described previously [23].

### Size exclusion analysis

To analyze the molecule weight of purified proteins, size exclusion chromatograph was performed using Superdex 200 3.2/300 (GE health) as described previously [25, 26]. Standard protein markers with different molecular weights (Sigma Aldrich, Cat# MWGF200) were used in this study.

### Cell culture

All the cell lines, including the Muc1-positive human colorectal cancer cell lines HT29 and LS174T, the human ovarian cancer cell line, SKOV3, the Muc1 negative human liver cancer cell line HepG2, and CHO cells, were obtained from the Type Culture Collection of the Chinese Academy of Sciences, Shanghai, China. CHO and LS174T was cultured in 1640 medium (Gibco, Life Technologies, China) with 10% FBS (Gibco, Life Technologies, USA) and 1% penicillin/streptomycin (Hyclone). HT29, SKOV3 and HepG2 were cultured in DMEM medium (Gibco, Life Technologies, China) with 10% FBS (Gibco, Life Technologies, USA) and 1% penicillin/streptomyciin (Hyclone). All cells were maintained at 37°C with 5% CO_2._ Human peripheral blood mononuclear cells (PBMCs) were prepared from healthy donors using Ficoll density centrifugation as described previously [23]. NK cells were then prepared from fresh isolated PBMCs using an EasySepTM Human NK Cell Enrichment Kit according to the manufacturer’s instructions (Stem cell Co. Ltd, Cat#19055). All healthy donors signed informed consent prior to blood sampling. This study was approved by the Research Ethics Committee of the Sun Yat-sen University.

### ELISA assay

For ELISA analysis, recombinant human Muc1 (Acrobiologicals) (0.1μg per well on 96 well plates) was coated overnight and then washed three times with PBS. Each well was then incubated with 200 μl blocking buffer (PBS+0.1%BSA) at 37°C for 1 hour to block non-specific binding. The plate was then washed three times. Muc1-Bi at 10^−3,^10^−2^,10^−1^,10^0^,10^1^,10^2^,10^3^ μg/ml was then added to corresponding wells. After incubation at 37°C for 1 hour, and washing for five times, HRP-conjugated anti-His antibody was added and incubated at 37°C for 35 minutes. After washing for five times, substrate was added and 450 nm reading was recorded and analyzed.

### Flow cytometry assay

For flow cytometry analysis, cells were collected followed by 2 washes with 1mL of ice-cold phosphate-buffered solution (PBS)+0.2% bovine serum albumin (BSA). The samples were then incubated with Muc1-Bi-1 or Muc1-Bi-2 (final concentration of 50 μg/ml, 1×10^5^ cells per sample) for 1 hour on ice. The cells were then washed twice and incubated with FITC-conjugated anti-His IgG (Abcam, Cat#Ab1206) for another 1 hour. Monoclonal antibody anti-Muc1 (Stem Cell Technologies, Canada, Cat # 60137) was used as positive control. After washing the cells three times, flow cytometry analysis was then performed (FC 500, Beckman Coulter).

### Western blot analysis

Western blot analysis was performed as described previously [26]. Briefly, HT29, LS174T, SKOV3, and CHO cells were collected and then lysed on ice for 30 min. 25 μg of total cell lysate was separated by 8% SDS-PAGE and transferred to PVDF membrane. The membrane was then incubated with the primary antibody (Muc1-Bi-1, Muc1-Bi-2 or α-tubulin) in 5% non-fat milk in TBST at room temperature for 1 h. After washing for three times, the membrane was incubated with secondary antibodies (anti-His HRP IgG or anti-mouse IgG rabbit) (Abcam, Cat# ab1187) at room temperature for 1 h. The signal was then detected by using Immobilon Western chemiluminescent HRP substrate (Merck Millipore, cat# WBKLS#500).

### Cytotoxicity assays

Cytotoxicity assays were performed as described previously [27, 28]. Briefly, tumor cells were seeded in 96-well plates at a density of 2,500 cells/well and were incubated at 37°C in a 5% CO_2_ humidified incubator for 6 hrs. NK or peripheral blood mononuclear cells (PBMCs) were then added at 25,000 cells/well. Different concentrations of proteins (varied from 0.0001 to 10 μg/ml) were then added. After 72 hr incubation, live cells were measured using Cell Counting Kit-8 reagent (Dojindo) according to manufacturer’s instruction. The cell survival rate was calculated as following formula: [(live target cells (sample)-medium)/(live target cells (control)-medium)]×100.

### In vivo tumor growth inhibition assay

Non-obese diabetic-severe combined immunodeficiency disease (NOD/SCID) mice (female, 18–22g) were purchased from the Vital River Laboratory Animal Technology Co. Ltd and then housed in the animal experiment center of Sun Yat-sen University under sterile and standardized environmental conditions (20–26°C room temperature, 40–70% relative humidity, and 12h light-dark rhythm).

In vivo tumor growth assays were performed in two different methods. In the co-transplantation model as described previously [29, 30], Muc1 positive LS174T cells were prepared and then mixed with freshly isolated PBMCs. The mixed cell suspension (1×10^6^ LS174T cells and 5×10^6^ human PBMCs) was then transplanted to the right flank of NOD/SCID mice by subcutaneous injection in a total volume of 200 μL per mice. One hour after transplantation, Muc1-Bi-1 and Muc1-Bi-2 antibodies (20 μg/mice or 5 μg/mice) or vehicle control (PBS) were administered intraperitoneally (i.p.) (n = 7 each group). Treatment was continued daily over the following 7 days. The weight of mice and the volume of tumor were measured daily. Tumor volume was calculated by the formula (width^2^×length)/2.

In another model, Muc1 positive LS174T cells were prepared and then transplanted to the right flank of NOD/SCID mice by subcutaneous injection in a total volume of 1×10^6^ LS174T cells, 200 μL per mouse. When the tumor volumes reach to 50–100mm^3^, fresh prepared human PBMCs(5*10^6^/mouse) were injected intraperitoneally. Bispecific antibodies were then administered intraperitoneally (i.p.) every 2 days (n = 6 each group). Mouse weights and tumor volumes were measured daily over the following 14 days. Tumor volume was calculated by the formula (width^2^×length)/2.

All animals were obtained from the Laboratory Animal Center of Sun Yat-sen University. All animal experiments were carried out with the approval of the Ethical Committee of Sun Yat-sen University. The animals were maintained under pathogen-free conditions and provided sterile food and water in accordance with the Guide for the Care and Use of Laboratory Animals of Guangdong Province. No extreme stress and sickness was observed during the experiments. In the end of experiments, the animals were euthanized by cervical dislocation with the approval of the Ethical Committee of Sun Yat-sen University.

### Data analyses

All data were expressed as mean ± standard deviations (SD) of triplicates from one representative of three independent experiments unless otherwise specified. A two-tailed Student’s t-test was used for the comparison of differences between two independent groups. P<0.05 and P<0.01 were considered statistically significant differences.

## Results

### Muc1-Bi bispecific antibodies can be produced and purified in E.coli

The Muc1-Bi-1 bispecific antibody was first constructed by fusing two single domain antibodies, anti-Muc1 VHH (GenBank: FJ799116.1) targeting Muc1 positive tumor cells, and anti-CD16 VHH, which binds CD16 on NK cells and recruits NK cells [23] (Fig 1A top sequence). A His_6_-tag was added at the c-terminal to facilitate Ni-NTA purification and detection. Though single domain antibodies have high homologous to human VH antibodies, humanization of single domain antibodies can potentially reduce immunogenicity. As humanization has been successfully done for camel single domain antibodies [24], the humanized Muc1-Bi bispecific antibody, Muc1-Bi-2, was generated by grafting the CDRs of anti-Muc1 VHH and anti-CD16-VHH to the framework of DP-47 with minimal changes (Fig 1A, bottom sequence). For both bispecific antibodies, the anti-Muc1 VHH and anti-CD16-VHH were linked by two amino acids, Glycine-Serine (Fig 1B), as the short linker does not affect single domain antibody functions.

As single domain antibodies are highly soluble and stable, cytoplasm expression in *E*.*coli* was used to produce both Muc1-Bi-1 and Muc1-Bi-2. Both Muc1-Bi bispecific antibodies are partially soluble and can be purified by Ni-NTA affinity purification (Fig 1C) with a yield of ~0.45mg/L.

To characterize the purified Muc1-Bi bispecific antibodies, size exclusion chromatography was performed to analyze the molecular weight of Muc1-Bi bispecific antibodies. Both Muc1-Bi bispecific antibodies ran as a single peak with a molecular size of approximately 29 kD, which was the expected size of Muc1-Bi monomers, suggesting that majorities of the Muc1-Bi-1 and Muc1-Bi-2 are in the form of monomer (Fig 1D). Thus, both Muc1-Bi-1 and Muc1-Bi-2 were further analyzed.

### Both Muc1-Bi-1 and Muc1-Bi-2 can bind Muc1 positive cells

**Fig 2:**
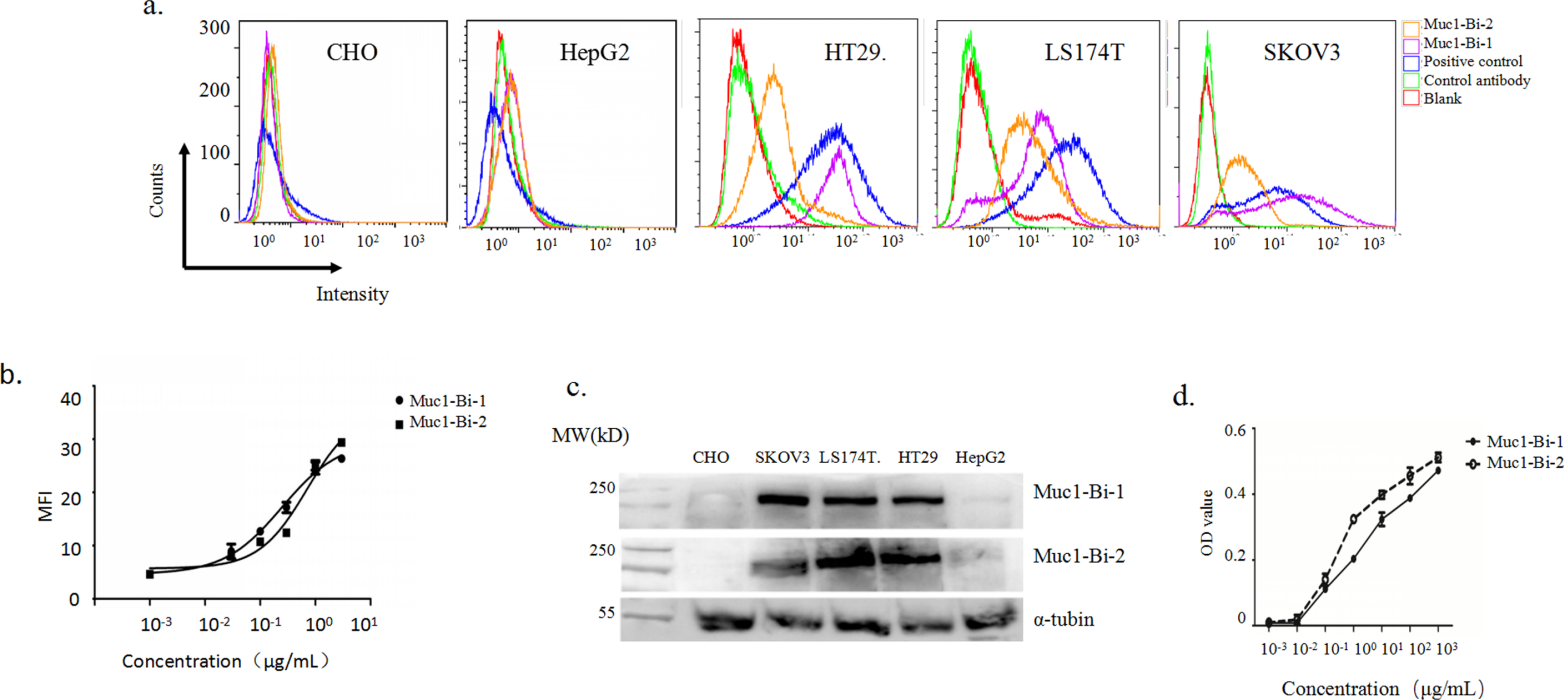
Muc1-Bi-1 and Muc1-Bi-2 can recognize Muc1 positive tumor cells. a). Flow cytometry analysis of Muc1-Bi-1 and Muc1-Bi-2 on Muc1-negative cell lines HePG2 and CHO, Muc1-positive cell lines, HT29, LS174T, and SKOV3. Red line indicates cells with no staining; green line indicates cells with only anti-His-FITC staining; blue line indicates cells with positive control anti-Muc1 antibody; purple line indicates cells with Muc1-Bi-1 protein; orange line indicates cells with Muc1-Bi-2 protein. b). Quantitative analysis of Muc1-Bi-1 and Muc1-Bi-2 binding of LS174T cells by flow cytometry. c). Western blot analysis of Muc1 expression in different cell lines using Muc1-Bi-1 (top) and Muc1-Bi-2 (bottom). d). ELISA analysis of Muc1-Bi-1 and Muc1-Bi-2 binding on recombinant huMuc1.

To check whether Muc1-Bi-1 and Muc1-Bi-2 can bind to Muc1-positive cells, flow cytometry analysis was performed using Muc1-positive cells, HT29, LS174T, and SKOV3, and Muc1-negative cells, HepG2 and CHO cells. For Muc1 positive cells, HT29, LS174T and SKOV3, Muc1-Bi -1 and Muc1-Bi-2 or commercial antibody showed positive staining (Fig 2A); but very low or no staining on Muc1 negative cells HepG2, CHO (Fig 2A), suggesting that Muc1-Bi-1 and Muc1-Bi-2 bind specifically to Muc1 positive cells. The binding of Muc1-Bi-1 and Muc1-Bi-2 to Muc1 positive cells were farther analyzed using SKOV3 cells by flow cytometry. Binding with different concentrations of Muc1-Bi-1 and Muc1-Bi-2 on SKOV3 cells were measured and analyzed (Fig 2B). The similar binding curve suggested that Muc1-Bi-1 and Muc1-Bi-2 have similar binding affinity to Muc1 positive cells (Fig 2B).

To further confirm the specific binding of Muc1-Bi-1 and Muc1-Bi-2, western blot analysis was also performed (Fig 2C). Consistent with flow cytometry analysis, both Muc1-Bi-1 and Muc1-Bi-2 can recognized Muc1 from HT29, LS174T and SKOV3 cells, but not HepG2 and CHO cells (Fig 2C). ELISA was also performed to check the binding of Muc1-Bi-1 and Muc1-Bi-2 on Muc1 using recombinant human Muc1 protein (Fig 2D). Both Muc1-Bi-1 and Muc1-Bi-2 can bind to Muc1 with similar rate (Fig 2D). those data suggest that Muc1-Bi-1 and humanized Muc1-Bi-2 maintained the binding affinity of anti-Muc1 VHH.

SPR analysis also confirmed strong binding of Muc1-Bi-1 and Muc1-Bi-2 to CD16 with a Kd of 10nM, similar to the Kd of anti-CEA-VHH/anti-CD16-VHH bispecific antibody [23], suggesting that humanized anti-CD16 VHH in Muc1-Bi-1 and Muc1-Bi-2 also maintained the binding affinity of anti-CD16 VHH.

### Muc1-Bi-1 and Muc1-Bi-2 mediate potent cytotoxic activities against Muc1 positive tumor cells

**Fig 3:**
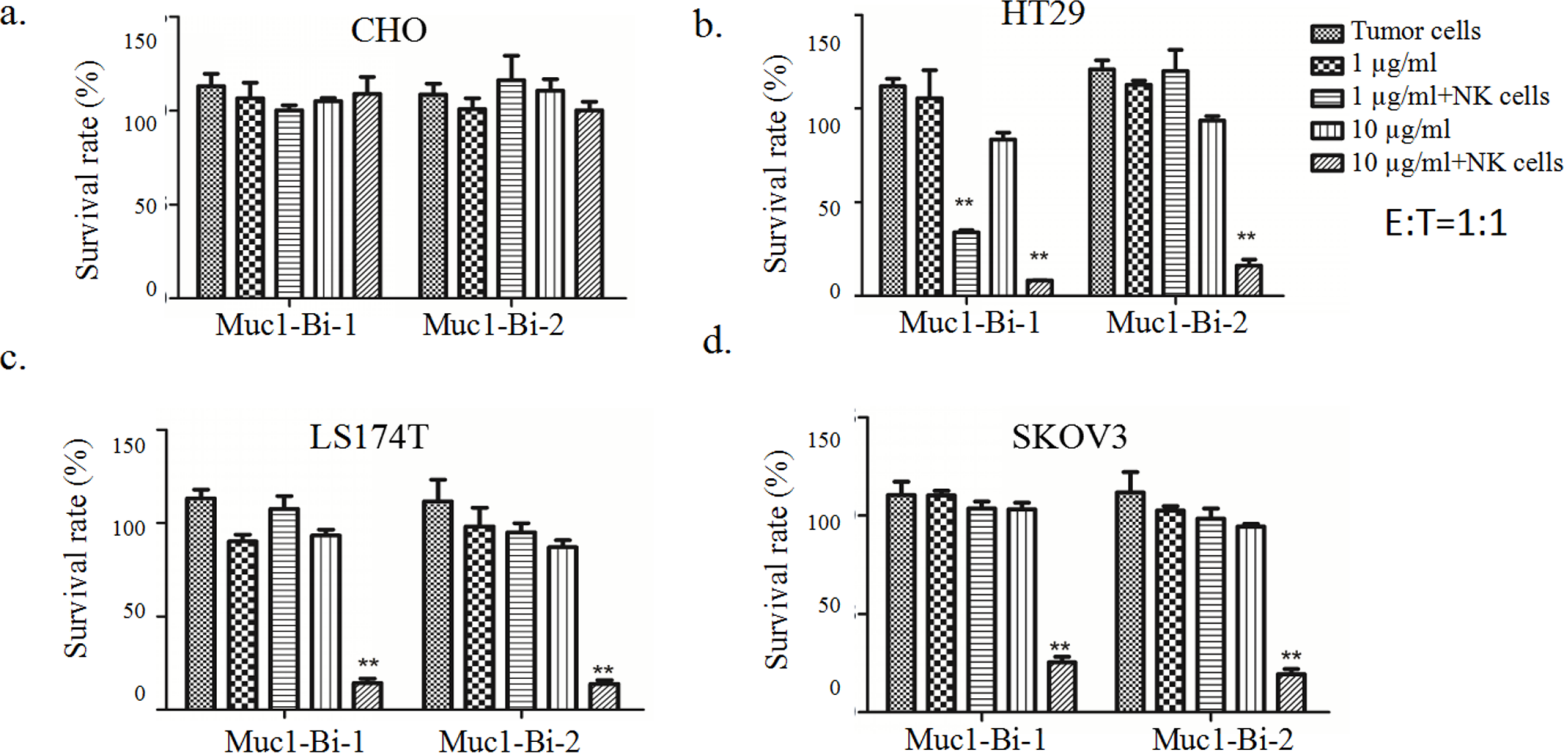
Muc1-Bi-1 and Muc1-Bi-2 mediate specific cytotoxic activities (E:T ratio = 1:1). Cytotoxicity assays were performed as described in the Materials and Method. Muc1-Bi-1 and Muc1-Bi-2 were incubated with different cell lines in the presence of NK cells. The effector cells (NK cells) (2500 cells/well) and target cells, CHO, HT29, LS174T, and SKOV3 (2500 cells/well) at ratio of 1:1 (E:T ratio = 1:1). Data are means ± SD, * P < 0.05; ** P < 0.01 compared with control.

**Fig 4:**
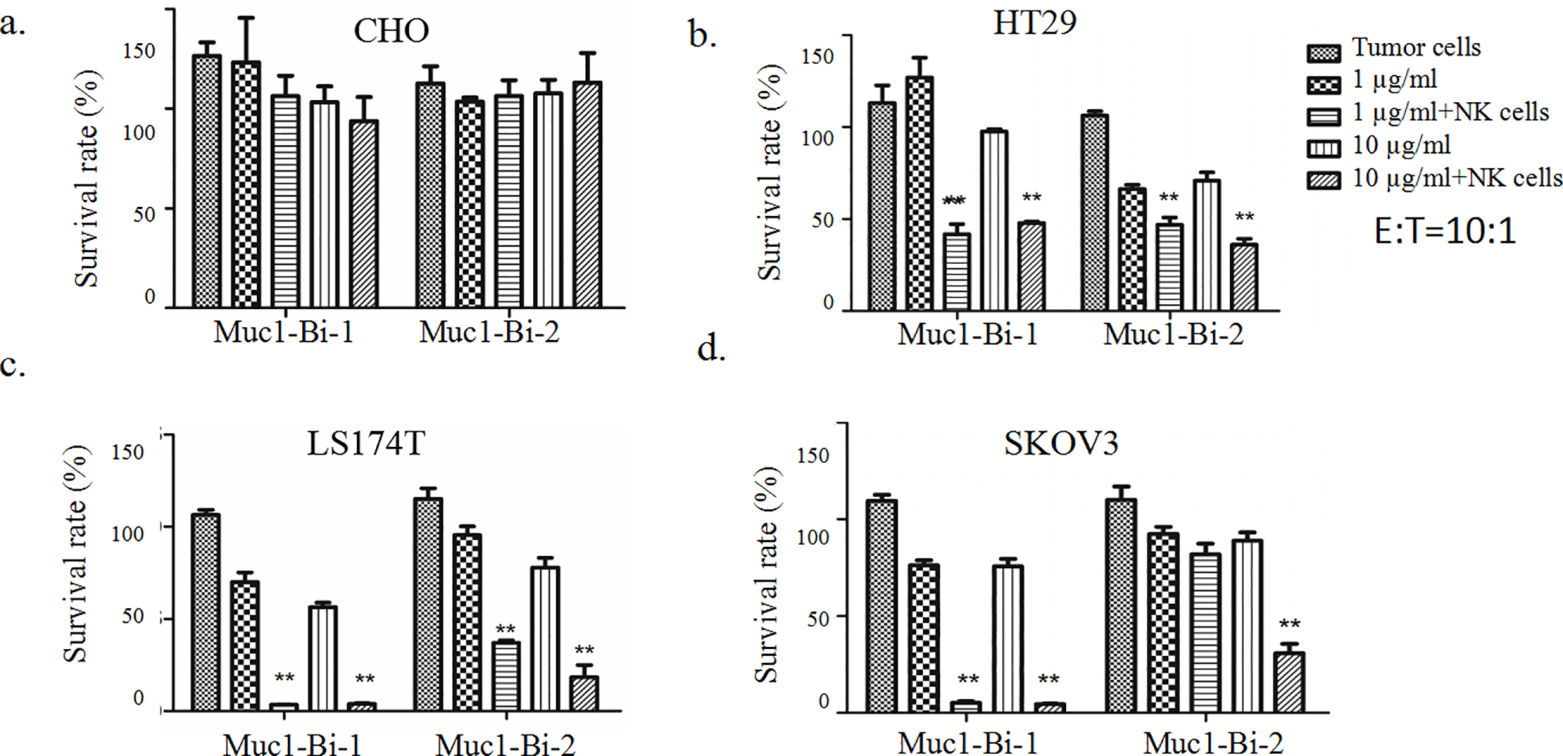
Muc1-Bi-1 and Muc1-Bi-2 mediate specific cytotoxic activities (E:T ratio = 10:1). Cytotoxicity assays were performed as described in the Materials and Method. Muc1-Bi-1 and Muc1-Bi-2 were incubated with different cell lines in the presence of NK cells. The effector cells (NK cells) (25000 cells/well) and target cells, CHO, HT29, LS174T, and SKOV3 (2500 cells/well) at ratio of 10:1 (E:T ratio = 10:1). Data are means ± SD, * P < 0.05; ** P < 0.01 compared with control.

**Fig 5:**
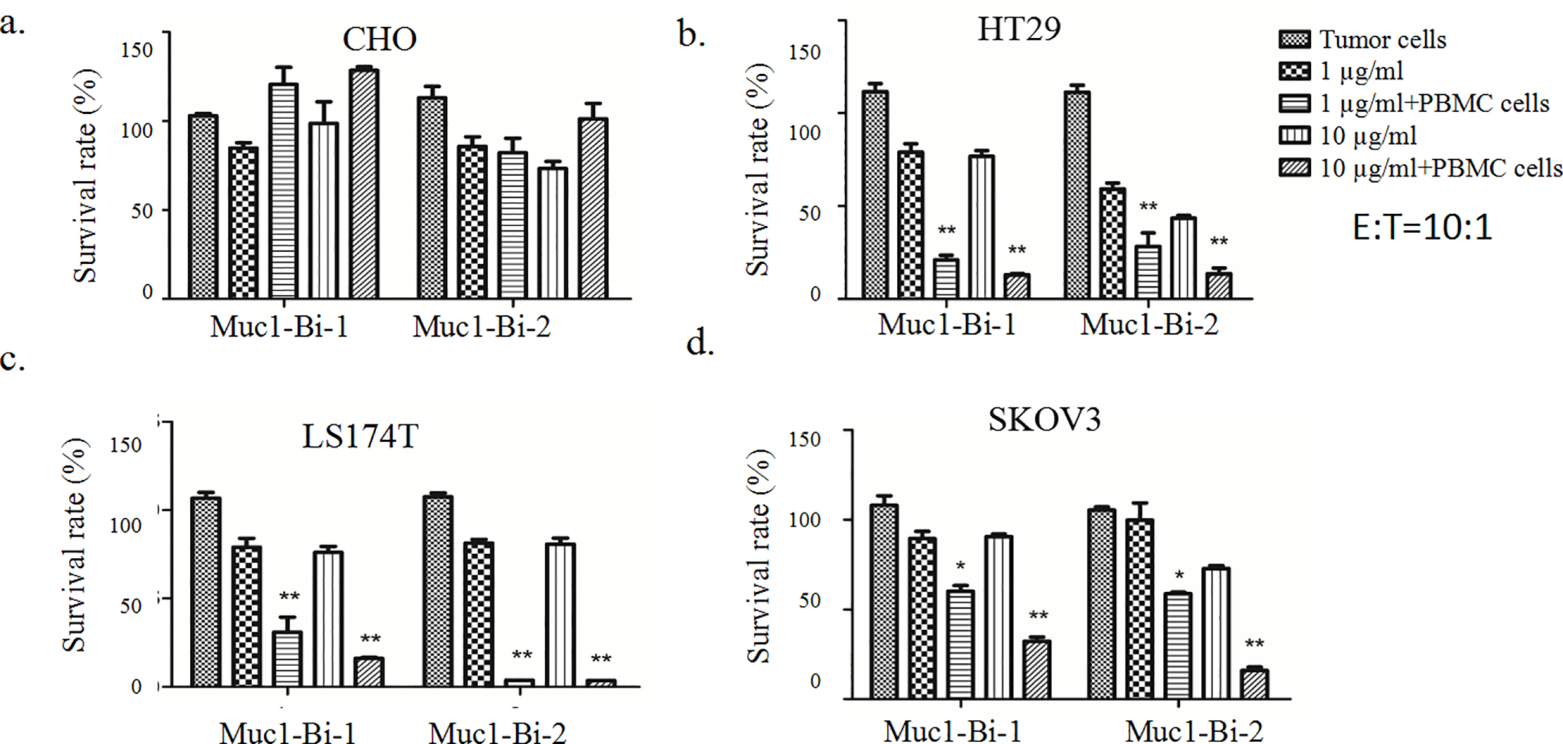
Muc1-Bi-1 and Muc1-Bi-2 mediate specific cytotoxic activities (E:T ratio = 10:1). Cytotoxicity assays were performed as described in the Materials and Method. Muc1-Bi-1 and Muc1-Bi-2 were incubated with different cell lines in the presence of PBMCs. The effector cells (PBMCs) (25000 cells/well) and target cells, CHO, HT29, LS174T, and SKOV3 (2500 cells/well) at ratio of 10:1 (E:T ratio = 10:1). Data are means ± SD, * P < 0.05; ** P < 0.01 compared with control.

To check whether Muc1-Bi-1 and Muc1-Bi-2 can medicate specific tumor cell killing, cytotoxic assays were performed using Muc1 positive cell lines, HT29, LS174T, SKOV3, and Muc1 negative cell line CHO. Different tumor cells were incubated with Muc-Bi-1 and Muc1-Bi-2 in the presence of NK cells or PBMCs (Fig 3). For Muc1 negative CHO cells, Muc1-Bi-1 had no cell killing activity either alone or in the presence of NK cells (Figs 3A and 4A), or PBMCs (Fig 5A). For Muc1 positive tumor cells, HT29, LS174T, or SKOV3, Muc1-Bi-1 alone had no cell killing activity at 1 μg/ml or 10 μg/ml (Figs 3B and 3C and 3D and 4B and 4C and 4D and 5B and 5C and 5D). In the presence of NK cells (Figs 3 and 4) or PBMCs (Fig 5), potent cell killing was observed. Higher cell killing was observed when NK or PBMC cells to the tumor cells ratio was 10:1 for both lower Muc1-Bi-1 concentration 1 μg/ml and higher Muc1-Bi-1 concentration 10 μg/ml (Figs 3 and 4 and 5). Similar to Muc1-Bi-1, Muc1-Bi-2 exhibited no cell killing for Muc1 negative CHO cells (Figs 3A and 4A and 5A), but potent immune cell dependent cell killing against Muc1 positive tumor cells (Figs 3B and 3C and 3D and 4B and 4C and 4D and 5B and 5C and 5D).

**Fig 6:**
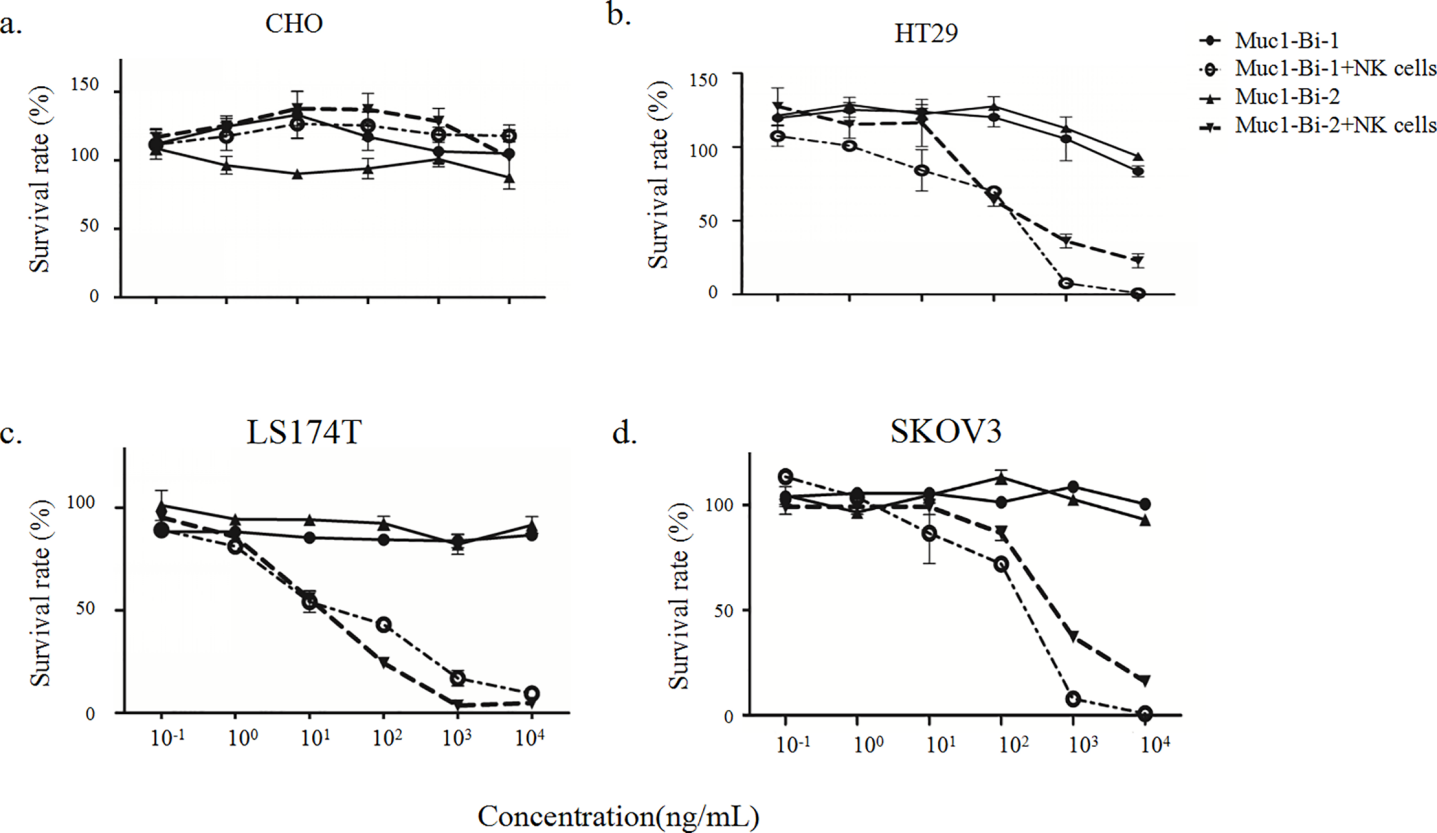
Muc1-Bi-1 and Muc1-Bi-2 mediate specific cytotoxic activities in a dose dependent manner. Cytotoxicity assays were performed as described in the Materials and Method. Muc1-Bi-1 and Muc1-Bi-2 were incubated with different cell lines with or without the presence of NK cells (E:T ratio = 10:1). a). CHO, b). HT29, c). LS174T, d). SKOV3). The concentrations of Muc1-Bi-1 or Muc1-Bi-2 were ranging from 0.1 ng/ml to 10 μg/ml.

The dose dependent cell killing by Muc1-Bi-1 and Muc1-Bi-2 was further analyzed using different concentrations of Muc1-Bi-1 and Muc1-Bi-2. No cytotoxic activity against CHO cells was observed regardless of the presence of NK cells or the concentrations of Muc1-Bi-1 and Muc1-Bi-2 (Fig 6A). For Muc1 positive cells, HT29 (Fig 6B), LS174T (Fig 6C), and SKOV3 (Fig 6D), increased cytotoxic activities were observed with increased concentrations of Muc1-Bi-1 and Muc1-Bi-2 in the presence of NK cells. These data suggested that the cytotoxic activity of Muc1-Bi-1 and Muc1-Bi-2 depends on the expression of Muc1 and the presence of NK cells.

### Muc1-Bi inhibits tumor growth in vivo

**Fig 7:**
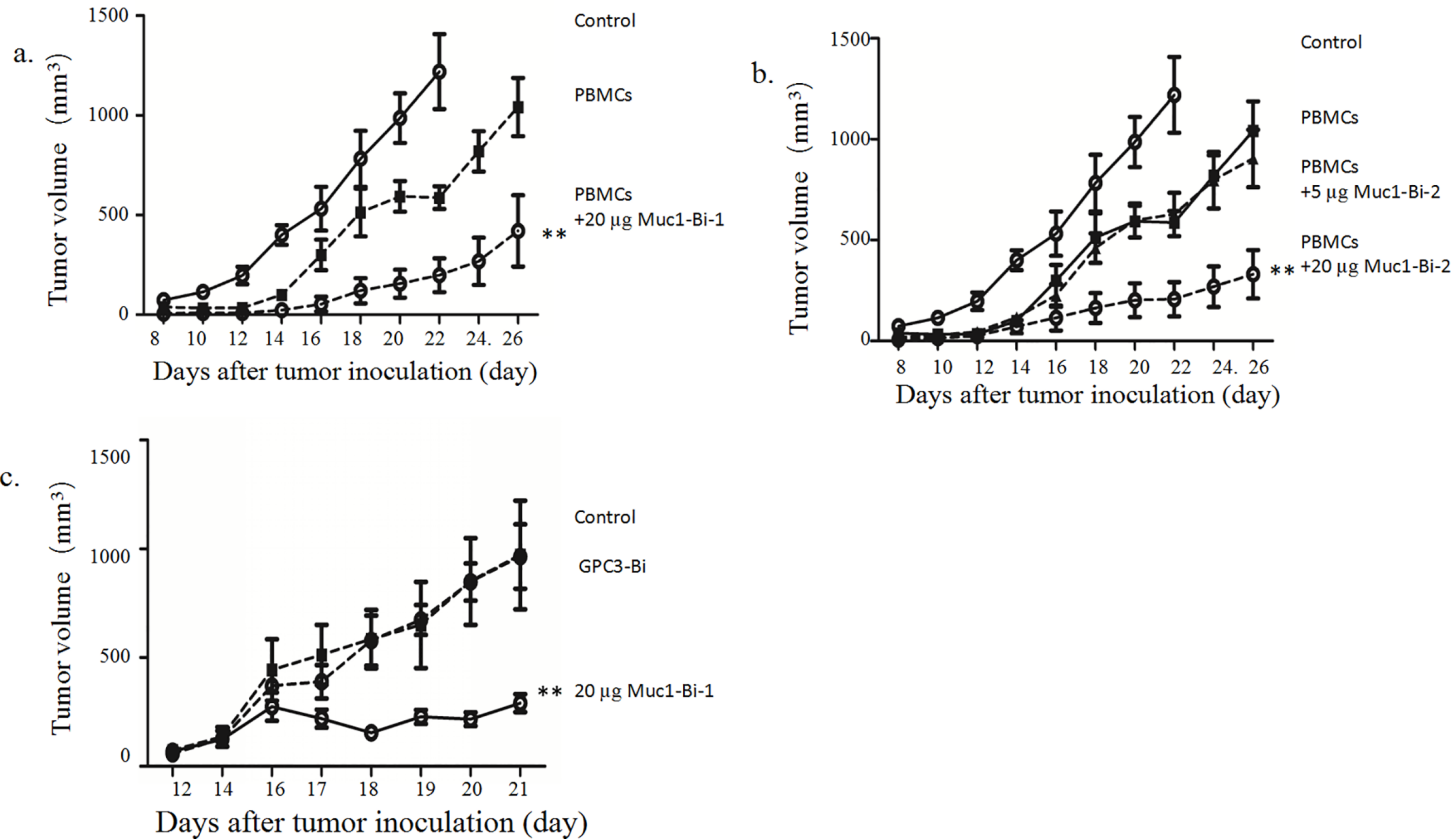
Muc1-Bi-1 and Muc1-Bi-2 inhibit tumor growth *in vivo*. NOD/SCID mice (n = 7/group, female) were engrafted subcutaneously with LS174T cells (1×10^6^ cells/mice) (circle, solid line) with or without freshly isolated human PBMCs (5×10^6^ cells/mice). The mice were then treated with Muc1-Bi-1 (20μg/mouse), or Muc1-Bi-2 (5 and 20μg/mouse) as described in the Materials and Methods. For the mice transplanted with LS174T and PBMCs simultaneously, a). LS174T only (circle, solid line), LS174T with PBMCs (square, dash line), LS174T and PBMCs treated with Muc1-Bi-1 (20μg/mouse) (circle, dash line). b). LS174T only (circle, solid line), LS174T with PBMCs (square, dash line), LS174T and PBMCs treated with Muc1-Bi-2 (5μg/mouse) (triangle, dash line), Muc1-Bi-2 (20μg/mouse) (circle, dash line). c). PBMCs were transplanted after tumor size reaches 50–100 mm^3^, PBS (circle, solid line), GPC3 (square, dash line), Muc1-Bi-1 (20μg/mouse) (circle, dash line). The data represent the average tumor volumes of 6 mice. Error bars represent the standard deviation (**P< 0.01, t test, Muc1-Bi-1 and Muc1-Bi-2 (20μg) *vs* the other groups).

To test whether Muc1-Bi-1 and Muc1-Bi-2 can suppress tumor growth *in vivo*, Muc1 positive tumor cells LS174T were grafted onto NOD/SCID mice together with freshly prepared PBMCs, partial tumor growth inhibition was observed with PBMCs alone (Fig 7A). When mice were also treated with Muc1-Bi-1 in the presence of PBMCs, significant tumor growth was observed (Fig 7A). Furthermore, tumor growth was observed in only 4 of 7 mice that were treated with 20 μg Muc1-Bi-1 protein. No tumor growth was observed in the other 3 mice, even 4 weeks after tumor cell transplant.

For Muc1-Bi-2 protein, similar results were observed (Fig 7B). significant tumor growth was observed when the mice were treated 20 μg Muc1-Bi-2 protein with PBMCs transplanted. Tumor growth was observed in only 5 of 7 mice that were treated with 20 μg Muc1-Bi-2 protein. No tumor growth was observed in the other 2 mice. Partial tumor growth inhibition was observed for mice treated with 5 μg Muc1-Bi-2 protein, indicting a dose response. These data suggested that Muc1-Bi-1 and Muc1-Bi-2 can inhibit tumor growth in the LS174T xenograft mouse model.

To further analyze the therapeutic effect of Muc1-Bi in vivo, LS174T cells were grafted onto NOD/SCID mice first. When the tumor sizes reach 50–100mm^3^, freshly prepared PBMCs were transplanted into mice. The mice were then treated with Muc1-Bi-1. Partial tumor growth inhibition was observed by Muc1-Bi-1 (Fig 7C). For the mice were treated with PBS alone, no significant tumor growth was observed (Fig 7C). The mice were also treated with the same molar amount of control bispecific antibody GPC3-bi, which recognizes GPC3 positive cells, in the presence of PBMCs. As LS174T cells have no GPC3 expression, no significant tumor growth was observed for GPC3-bi treatment (Fig 7C). These data confirmed that Muc1-Bi-1 can inhibit Muc1 positive tumor growth *in vivo*.

## Discussion

Immunotherapy has been proving as a promising cancer therapy with superior efficacy and less toxicity than many other current cancer therapies [31]. Among the different approaches for cancer immunotherapy, bispecific antibodies have been intensively investigated, especially bispecific antibodies engaging T cells. Blinatumomab, a tandem ScFv bispecific antibody, has already been approved for the treatment of B-cell leukemia [32]. Many other bispecific antibodies are also in different stages of development [33].

Muc1 have been attracted much attention as a cancer immunotherapy target as it is frequently overexpressed in many types of cancers, including colon, lung, pancreas, breast, ovarian, prostate, kidney, stomach and head and neck cancers [14–16]. Moreover, the difference of glycosylation between tumor Muc1 and normal tissue Muc1 presents a nice distinction between tumor cells and normal cells. A variety of approaches have been tested, including CAR-T [21]. However, bispecific antibody approach has not been reported for Muc1. In this study, we generated Muc1 bispecific antibody Muc1-Bi and tested its function for Muc1 overexpression cancer therapy. We used single domain antibodies instead of single-chain Fv fusions to construct Muc1-Bi bispecific antibodies as single-chain Fv are generally less stable, have the tendency to aggregate, and also difficult to be expressed in bacteria[34, 35]. Single domain antibodies or VHH were derived from the natural camel heavy-chain only antibodies, and lack a light chain and the first constant domain (CH1) [35]. Compared with single-chain Fv, single domain antibodies have lower molecular weight (~15kd), increased stability, and better solubility in *E*.*coli*.

To engage Muc1 tumor cells, anti-Muc1 VHH[36], which was raised against tumor derived Muc1 proteins, was used to target Muc1 tumor cells. To engage immune cells, we used anti-CD16 VHH to engage NK cells [33]. To reduce the immunogenicity of single domain antibodies, we also humanized Muc1-Bi bispecific antibody by grafting the CDRs of anti-Muc1 VHH and anti-CD16-VHH to the framework of DP-47 with minimal changes (Fig 1A), which has been used previously to humanize camel single domain antibodies [24]. Both Muc1-Bi bispecific antibodies can be expressed and purified from E. coli with good solubility and stability, likely due to their lower molecular weight and generally more stable than conventional scFv [34]. Using different approaches, including flow cytometry, western blot and Elisa, both Muc1-Bi bispecific antibodies can bind Muc1 positive cells specifically. Muc1-Bi bispecific antibodies also had potent specific cytotoxicity against Muc1 positive cells. In vivo studies also demonstrated that Muc1-Bi bispecific antibodies can inhibit tumor growth in the presence of PBMCs in NOD/SCID mice.

The studies in this work confirmed that Muc1-Bi bispecific antibodies have potent anti-tumor activity against Muc1 positive tumor cells. Compared to Muc1-Bi-1, the humanized Muc1-Bi-2 showed similar in vitro and in vivo activities. Considering its potential lower immunogenicity, Muc1-Bi-2 presents as a valid molecule to combat Muc1 positive tumors in clinic. The lower molecular weight of Muc1-Bi bispecific antibodies may have the disadvantage of more rapid clearance in vivo. However, the lower molecular weight of Muc1-Bi bispecific antibodies may penetrate tumor tissue better. To enhance its potential in clinic, conjugation with PEG or other means to increase in vivo half-life may be considered for Muc1-Bi-2. Besides NK cells, recruiting other immune cells, such as T cells using anti-CD3, can also be explored for bispecific antibodies targeting Muc1.
